# A Plea for Consistency in the Use of Terms Applied to Local Remedies Classified According to Their Pharmacological Action

**Published:** 1909-07-15

**Authors:** E. T. Loeffler


### A PLEA FOR CONSISTENCY IN THE USE OF TERMS APPLIED TO LOCAL REMEDIES CLASSIFIED ACCORDING TO THEIR PHARMACOLOGICAL ACTION.

BY E. T. LOEFFLER, B.S., D.D.S.

Read before the First District Dental Society, March 11, 1909.

Consistency or uniformity in theory and practice has been urged in every branch of the Dental Profession. Uniformity in the practice of crown and bridge work; uniformity in the treatment and extirpation of the pulp; uniformity in the practice of orthodontia; and uniformity in the preparation and filling of cavities.

The question of consistency or uniformity in the use of terms is equally important. There is nothing, it seems to me, that reflects so much upon the dignity of a profession as the haphazard use of terms. The last edition of the United States Pharmacopeia gives a number of changes in the names of drugs. The reason for this is quite apparent; it is to bring about a greater degree of uniformity.

By consistency I mean an agreement with itself at different times. "Consistency in use," is the phrase to which I wish to call your special attention. When a term or word has been defined in a certain way, its use or application should always be made with due respect to this definition. Definitions, of course, are more or less arbitrary, but even when a term has been made, as it were, to mean a certain thing, this meaning or definition should be kept in mind when a practical application is made.

Let us take, for example, the word pharmacology. According to Professor Cushny, pharmacology is, "A study of changes induced in living tissues by the administration, in a state of minute division, of such unorganized substances as are not merely used as foods." I very much doubt the existence of any definition in all medical science that will compare with this for comprehensiveness and is so lacking in hyperbole. There are several other terms, as physiology, pathology, bacteriology, etc., that could have a similar definition.

Pharmocology might, with propriety, be considered a department of biology, and is, as has been suggested, quite closely related to other sciences included under that head.

As indicated in Cushny's definition, the drug must be introduced from without, because so many active agents are formed within the body itself that a study of the changes they induce belongs more appropriately to the department of physiology; on the other hand, an investigation of the effects produced by organized bodies introduced from without should be included under the head of bacteriology.

The same consistency ought to be maintained in the use and application of other terms. Since the dentist is specially interested in topical remedies, I shall endeavor to confine my discussion to pointing out the lack of uniformity that exists in the application of terms generally used for such purposes.

As a first example of inconsistency, let me call your attention to the three terms, caustic, escharotic and corrosive. Dorland, in his new Medical Dictionary, defines them in the following manner: "Caustic. (1) Burning or corrosive; destructive to living tissue; (2) Having a burning taste. (3) An escharotic or corrosive agent. Escharotic. (1) Corrosive; capable of producing an eschar. (2) A corrosive or caustic agent. Corrosive. Destructive to the texture or substance of the tissue."

Here we can readily notice the great similarity of meaning—how one word is defined in terms of the other. They are to all intents and purposes synonymous. Webster says, "If no words are synonymous except those which are identical in use and meaning, so that one can, in all cases be substituted for the other, we have scarcely ten such words in our language. Words may thus coincide in certain connections, and so be interchanged, when they cannot be interchanged in other connections."

The drugs and agents usually classified under the above heads differ so materially in their nature and action that it would seem wise to give each word used to designate them a different meaning.

To explain in detail this difference in action I cannot do better than quote from Steven's latest edition of Materia Medica and Therapeutics. He says, "Escharotics are agents that corrode or disorganize the tissues. Some, like arsenic, have a specific poisonous action upon the cells; some, like the strong mineral acids, extract water from the tissue and precipitate the proteids; some, like the alkali hydrates, not only extract water but dissolve the proteids and form with them soluble compounds; some, like nitrate of silver, form with the proteids insoluble albuminates and, in so doing, liberate an acid; some, like nitrate of mercury, poison the cells directly, form albuminates with their proteids and also set free an acid; while others still, like bromin, are powerful oxidizers of organic matter."

By the profession in general and in most text-books, the terms, caustic, escharotic and corrosive are used interchangeably to express these different actions as stated above. It does seem very reasonable and fair, that by slightly changing the meaning of each of the above terms, we could, at least, form three distinct groups of the agents usually classified under this head.

After considerable thought and study I have concluded to adopt the following definitions, which I trust will receive your careful consideration.

A caustic is a drug or agent that coagulates albumen, is more or less limited in its action and forms a soluble eschar, as phenol and trichloracetic acid.

An escharotic is any drug or agent that coagulates albumen, is limited in its action, and forms a more or less insoluble eschar, as nitrate of silver and the actual cautery.

A corrosive is any drug or agent that may or may not coagulate albumen and is more or less unlimited in its action, as the alkaline hydrates and the stronger mineral acids.

These definitions give each term a distinct meaning and refers to a particular class of agents. Expressions, therefore, like "lunar caustic," and "caustic soda," should be considered misnomers. Both the drugs referred to differ quite materially in their action, and the term caustic, according to the above definitions, should not be used in either case.

Again, each of these three classes of agents, as suggested, has a distinct practical application. Caustics, for example, as phenol and trichloracetic acid, are used very largely for removing hypertrophied gum tissue, as in the preparation of proximal cavities and the setting of Logan crowns. In such cases deep penetration or an insoluble eschar is not desired. If, on the other hand, we wish to produce an insoluble eschar, as in the treatment of cancer sore or check hemorrhage, we use an escharotic like nitrate of silver or the actual cautery. These agents form a more or less insoluble eschar and will remain intact for some time.

In case we wish to remove a small tumor like a wart, a corrosive, like nitric acid is indicated, because both caustics and escharotics are too limited in their action to produce the desired effect.

Long, in his text-book on Materia Medica, Therapeutics and Prescription Writing, classifies all these agents under the head of escharotics, although, in the introductory remarks of the chapter, he clearly intimates that a division should be made.

Right in this connection I would like to state that it has always been a difficult matter to classify arsenous trioxide. Long says, "It stands alone in its characteristics as an escharotic. It cannot be called an irritant. It is not a corrosive. It has no decided chemical affinities; therefore it is not escharotic by means of any apparent chemical action. It stands by itself as a vital or alterative escharotic, in that it acts only after being absorbed by the tissue elements, altering or destroying their vital processes in an obscure manner."

Now, while this may be true in regard to arsenous trioxide, most of the other drugs coming under this head can be very readily designated as belonging to a distinct class, as outlined above. The three terms, caustic, escharotic and corrosive are in use, why not apply them with a distinct meaning?

My experience in teaching the subject of Dental Therapeutics has been that students are continually failing to grasp the true meaning of definitions. They are after facts rather than principles, and text-books as well as instructors are at fault in not pointing out the difference and importance of clearer definition of common terms.

I am thoroughly convinced that a consistent application of technical terms is, perhaps, one of the most interesting and important features in all medical and dental science. This statement is true, to some extent at least, in every science; but with the progress of modern aseptic surgery, the amount of discrepancy in regard to the signification and use of certain terms is perfectly amazing as compared with the general intelligence on this important subject.

I have made a careful and conscientious study of this subject, and I very much doubt whether a better and clearer verification of the above fact can be given than is illustrated by the usual application of the terms, antiseptic, disinfectant, germicide and sterilize.

Professor Novy, of the University of Michigan, defines the terms referred to as follows:

"An antiseptic is any drug, chemical substance or agent that will retard or inhibit the growth or development of micro-organisms."

"A germicide is any drug, chemical substance or agent that will, in a limited time, destroy all forms of vegetative bacteria."

"A disinfectant is any drug, chemical substance or agent that will, in a limited time, destroy all active forms of pathogenic bacteria and their noxious products."

"To sterilize means to destroy all active forms of bacteria as well as spores."

It may seem like an imposition upon your good nature, but I cannot help giving you an unusual amount of repetition to make plain the distinction between such terms as antiseptic and germicide. In Professor Novy's laboratory book on bacteriology, we have the following statement: "The first action of a chemical substance, when added to a recently inoculated culture medium, is to inhibit the growth of the organism. If the chemical substance is highly poisonous, and is present in sufficient amount, it will evidently kill the bacteria present. On the other hand, many weak substances, commonly designated as preservatives, will prevent the development of bacteria, but are not able to destroy them.

Here we certainly have a clear distinction between the words antiseptic and germicide, the one destroys while the other only retards or inhibits. When sufficiently diluted, a germicide will, in most instances act as an antiseptic.

In his text-book on the principles of bacteriology, Professor Abbott points out very clearly the difference between the words sterilization and disinfection.

He says, "In most laboratories it is customary to employ the term sterilization for the destruction of bacteria by heat, and the term disinfection for accomplishing the same end by chemical agents. We shall endeavor to show that this distinction in the use of the terms is not strictly correct.

Those of you, who are familiar with such methods will remember that the use of the term sterilization for destroying bacteria quite likely arose from the fact that all culture media and other articles to be rendered free from bacterial life are not treated by chemical agents as a rule, but are exposed to heat in an apparatus known as a sterilizer. This process is called sterilization. On the other hand, however, undesirable articles, useless cultures and all infected material may be subjected to the action of chemical agents, called disinfectants, and the process is called disinfection.

By careful study and investigation I am thoroughly convinced that the term sterilization really means a complete destruction of all bacterial life, and may be accomplished either by thermal or chemical agents. Disinfection does not necessarily mean destruction of all living forms, but only those that are infectious. Both, however, may be accomplished by the same means.

An indefinite number cf illustrations might be given to show that, for example, an antiseptic agent simply retards or prevents, a germicide destroys all active forms, while a disinfectant is used principally to destroy pathogenic forms and their noxious products, and lastly, that sterilization means the destruction of all life.

Dr. Miller, in his work on "Micro-organisms of the Human Mouth," makes the statement that, "A solution which devitalizes spores in one minute is out of the question, and in fact, is not at all necessary, since the conditions which lead to the formation of spores do not exist in the mouth where we find almost exclusively the vegetative forms." Yet, in several instances, he uses this very term when the word "germicide" or "disinfectant" would have been more appropriate.

Some drugs, like phenol and the essential oils, may be used either as simple antiseptics or germicides by simply varying the strength of the solution. Others, however, like permanganate of potash and hydrogen peroxide, are excellent disinfectants and germicides, but are absolutely useless as simple antiseptics on account of their transitory character.

The real difference between the above agents is often best explained by giving their essential qualities. I shall give only one illustration, and that is in the case of a simple antiseptic: A desirable antiseptic should have the following qualifications: (1) It must have staying or lasting qualities or be only slightly soluble, otherwise it will be too rapidly absorbed and its action lost; (2) It must be non-irritating, because it would remain in contact with the tissues a long time; (3) only slightly toxic, on account of the danger of absorption; (4) it should not discolor the teeth; and, (5), it should have a pleasant taste and odor.

"Local anesthetic" is another expression that should not be used, inasmuch as we have another term that expresses that condition. Anaesthetics are usually defined as agents that abolish all insensibility. Analgesics, on the other hand, act locally and are used either to prevent or relieve pain. Anodynes are drugs or agents that are used to relieve pain, and not to prevent it. Obtundants are drugs or agents employed to desensitize sensitive dentine.

These terms have been coined for a special purpose, as it were, and yet how often do we hear or see in print such expressions as "local anaesthetic" instead of "local analgesic" or simply "analgesic;" and the word, "anodyne" for "analgesic" or "anaesthetic." Each of the above terms has a distinct use for which it was coined and intended.

We often fail to make ourselves understood or to make things plain, not from a desire to withhold information, but rather from an unintentional failure to use the proper words.

In conclusion, I would like to add that this subject has been to me a very interesting field for investigation for the past four or five years.

I sincerely trust that the task I have undertaken to point out, the imperative necessity of putting into practice the subject of my plea and of finding a way of doing it, may receive your hearty co-operation.

The probable result and the benefit that may be derived therefrom warrants, I think, not only the slight effort to endorse, but to put it into actual practice as well.

